# Porta hepatis in relation to portal vein among Indians

**DOI:** 10.6026/97320630018630

**Published:** 2022-07-31

**Authors:** Mrinalini Gaikwad, KN Geetha, K Prabhakaran, Gnanadesigan Ekambaram

**Affiliations:** 1 Department of Anatomy, Nootan medical college and Research Centre, Sankalchand Patel University, Visnagar - 384315, Gujarat, India; 2 Department of Anatomy, Malabar medical college and Research Centre, Ulliyeri, Kozhikode - 673315, Kerala, India; 3 Department of Physiology, Nootan medical college and Research Centre, Sankalchand Patel University, Visnagar - 384315, Gujarat, India

**Keywords:** Porta hepatis, portal vein, bile duct, hepatic artery, liver specimens

## Abstract

The porta hepatis / hilum of liver is a transverse fissure located in the inferior surface, where the major vessels and ducts enter or leave the organ. The major structures traversing the porta hepatis are the portal vein, the hepatic artery and the hepatic duct. Porta hepatis is an area of surgical and radiological significance. The knowledge of variations in structures traversing the porta hepatitis will reduce the risk of surgeries involving this area. Study was conducted in the department of anatomy dissection lab after obtaining ethical clearance. 30 liver specimens were used for these studies which were removed from the cadaver during under graduate teaching. Anatomical knowledge of variations in relations of structures present in porta hepatis is of immense help to surgeons and radiologists when they engage patients for clinical procedures like liver transplant, cholecystectomy and diagnostic procedures. Hence this study was aimed to observe the relations of portal vein in porta hepatis.

## Background:

The liver is the largest gland and the most important organ in human body [1]. It also performs an astonishingly large number of tasks that impact the whole body system. As the liver is involved in majority of the metabolic activities in the body, it is more likely to get affected by various pathological conditions [2,3]. A liver surgery poses great challenge even for expert hands. Hepatobiliary surgery requires comprehensive knowledge of anatomy of liver and surrounding structures [4]. Porta Hepatis (PH) is a nonperitoneal deep fissure on the inferior aspect of liver which acts as a gateway for entry or exit of neurovascular structures. These structures include hepatic artery surrounded by autonomic plexus of nerves and the portal vein entering the liver, whereas hepatic ducts and some lymphatics emerge out of the liver. Porta hepatis is bounded by left lobe, caudate lobe, caudate process, right lobe, fossa for gall bladder and quadrate lobe of liver. Due to presence of these key structures in a small area, the clinical procedures around this region are associated with many iatrogenic complications. Porta hepatis transmits important neurovascular structures and hence it acquires great importance while carrying out various surgical and radiological procedures [5-7]. Knowledge and variations of hepatic system is essential to minimize morbidity encountered during hepato-biliary surgeries [8]. Therefore, it is of interest to evaluate the relation of portal vein with structures at porta hepatis. These findings will be of significance for anatomists, surgeons operating on this region, and radiologists to avoid iatrogenic complications.

## Materials and Methods:

### Study place:

The study was carried out in the department of anatomy at medical College, Kamothe, Navi Mumbai.

### Study design:

This is a cross sectional, Observational study.

### Duration of study:

January 2011 to January 2012

### Sample size:

30 human liver specimens irrespective of age and sex were taken for the study.

### Inclusion criteria:

Intact and undamaged liver specimens were taken for the study

### Exclusion criteria:

Bodies on which abdominal surgeries was performed were excluded.

After acquiring institutional ethical committee clearance (MGM/IHS/RS/2011/1027) the study was carried out. The method of dissection was done according to the Cunningham's manual of practical anatomy volume - II [9]. By using dissection instruments (Figure 1 - see PDF) the liver specimens were removed and preserved in 10% formalin during regular dissection for medical undergraduate. Porta hepatis were identified and carefully dissected and studied. The structures at porta hepatis were identified and their relations to the portal vein were observed and noted down. Variations found were meticulously cleaned and photographed.

## Results:

Normal Relations at Porta Hepatis were observed in 29 liver specimens (Figure 2 - see PDF). To the left of portal vein is the left hepatic artery (This relation shows slight changes depending on the level of section of porta hepatis) if the section is through the upper part of porta hepatis. The left hepatic artery lies on the left side of portal vein, where as if the section is through the lower part the artery was observed anterior and to the left of portal vein. Figure 2(see PDF) Shows Normal relations at porta hepatis: Porta hepatis is situated between caudate lobe and quadrate lobe. The relations at porta hepatis are anterior to the portal vein are the common bile duct and Posterior to the portal vein is caudate lobe of liver. Figure 3(see PDF) Shows Variation at porta hepatis: Variation found only in one liver specimen (1 out of 30 ie. 3.3%). The variation is anterior to portal vein the common bile duct. Posterior to the portal vein inferior vena cava and Left side hepatic artery and also two arteries and two ducts were observed in the same specimen.

## Discussion:

Porta hepatis acts as the gateway of liver through which important structures enter and exit, namely the Portal vein, hepatic artery, and hepatic duct. The anatomical arrangement of these structures is very important for surgeons and radiologists. Present study was conducted on 30 formalin preserved liver specimens. Structures at Porta hepatits were identified and their relations to portal vein were observed and noted. Variation found only in one liver specimens (1 out of 30) Anterior to portal vein the common bile duct, Posterior to the portal vein inferior venacava. Two arteries and two ducts were observed in this specimen in the left side hepatic artery. Normal relations were observed in all 29 liver specimens. Porta hepatis is situated between caudate lobe and quadrate lobe. The relations at porta hepatis are Anterior to the portal vein the commom bile duct, Posterior to the portal vein the caudate lobe, Left side of portal vein the left hepatic artery. Dhanalaxmi D et al. [3] studied the dimensions of Porta hepatis which included the transverse and anteroposterior diameters and the circumference which showed a wide variation. The number of structures and their arrangement with various combinations was observed, the most common combination observed was of two veins, two arteries, and one duct which were seen in 36% of specimens. The mean transverse diameter, anteroposterior diameter, and total circumference of PH were 3.17 ± 0.50, 1.68 ± 0.36, and 10.46 ± 1.415 cm, respectively. Eighteen specimens showed presence of two arteries, two veins, and one duct at PH. Maximum number of arteries, veins, and ducts passing through PH were 5, 4, and 1, respectively. The ducts were anterior, arteries in the middle, and veins were posterior in PH of all the livers. Dimpy Gupta et al. [4] studied the Structures passing through porta hepatis, they observed high variations in terms of their number and combination. Most common combination of structures was observed by them were 2 arteries, 1vein and 1duct which was seen in 32% of specimen, followed by combinations of 3 arteries, 1 vein, 1 duct and 4 arteries, 1 vein and 1 duct in 16 % of liver specimens each. Another study conducted by M. Sapna et al. [1] found portal vein was the posterior most structure and the hepatic duct was the anterior most structure in all the liver they have studied. The hepatic artery and its branches were in between the duct and the vein. The number of ducts and vein found in porta hepatis varied 1 to 3 and the artery it was 1 to 4. In 51% of livers specimens, only one vein and in 80% of cases only one duct passed through the porta hepatis. In 56% of cases two arteries passed through the porta hepatis. Rela et al. [10] studied thirty seven consecutive livers specimens during ex vivo liver splitting procedure and reported an abnormal right accessory artery arising from the left hepatic artery was high in the porta hepatis. In Present study one liver specimen showed 2 arteries and 2 ducts and one vein, where the portal vein was posteriorly related to inferior vena cava. With the advent and routine use of multicolour computed tomography, three dimentional imaging of structures at porta hepatis is possible and the variations and anomalies are coming into notice more and more. Liver specimens studied have shown anatomical variations of structures at porta hepatis. Advancement in liver transplant and other biliary system surgeries increases the significance of variations of structures at porta hepatis.

## Conclusions:

Data shows that its knowledge will be of great importance to anatomists, surgeons, and radiologists.
